# Changes in Cranial Shape and Developmental Quotient at 6 Months of Age in Preterm Infants

**DOI:** 10.3390/children10050855

**Published:** 2023-05-11

**Authors:** Aya Nakanomori, Hiroshi Miyabayashi, Yukari Tanaka, Taishin Maedomari, Chihiro Mukai, Katsuya Saito, Aya Okahashi, Nobuhiko Nagano, Ichiro Morioka

**Affiliations:** 1 Department of Pediatrics and Child Health, Nihon University School of Medicine, Tokyo 173-8610, Japan; 2 Department of Pediatrics, Kasukabe Medical Center, Saitama 344-8588, Japan

**Keywords:** dolichocephaly, development, plagiocephaly, preterm infant, three-dimensional scanner

## Abstract

The purpose of this study was to investigate changes in cranial shape among preterm neonates aged 1–6 months and the relationship between developmental quotient (DQ) and cranial shape at 6 months of age. Preterm infants who were hospitalized in our hospital were prospectively followed for 6 months. The cephalic index (CI) and cranial vault asymmetry index (CVAI) were evaluated at 1 (T1), 3 (T2), and 6 months (T3) of age and compared with those of the full-term infants. The relationship between CI or CVAI and DQ at T3 was analyzed using the Enjoji Scale of Infant Analytical Development. A total of 26 participants born at 34.7 ± 1.9 weeks of gestation were included. The CI increased with age (T1: 77.2%, T2: 82.9%, T3: 85.4%, *p* < 0.01). The prevalence of dolichocephaly at T3 did not significantly differ from that in full-term infants (15.4% vs. 4.5%, *p* = 0.08). CVAI did not significantly differ between preterm and full-term infants. The DQ showed no significant correlation with either the CI or CVAI (correlation coefficients: 0.23 for CI, −0.01; CVAI). Dolichocephaly improved over time in preterm infants and no relationship between cranial shape and development was observed in preterm infants at 6 months of age.

## 1. Introduction

Various studies conducted since the 1970s have shown that the craniums of preterm infants are longer than those of full-term infants [1]. With advancements in neonatal intensive care unit (NICU) medical care and preterm infant survival, the prevalence of dolichocephaly has increased. Recent studies using three-dimensional (3D) scanners have shown that the craniums of preterm infants extend from birth to term-equivalent age [2], and that more than half of them have moderate or severe dolichocephaly at discharge or term-equivalent age [2,3,4]. In clinical practice, dolichocephaly can be observed in preterm infants beyond a few years. However, it was reported that dolichocephaly may improve at approximately 1 year of age [5,6,7].

Furthermore, preterm infants have been reported to have a higher risk of plagiocephaly than full-term infants [3,8,9]. Negative developmental effects of deformational plagiocephaly (DP) in full-term infants have been reported. At 36 months of age, children with a history of DP scored lower than children without DP on all scales of the Bayley score [10,11]. It was also reported that no infant with DP displayed accelerated growth [12]. However, there are only a few reports on the association between dolichocephaly and development in preterm infants [4]. The problem regarding cranial shape in preterm infants has been known for a long time but remains difficult to elucidate.

Using a 3D scanner, we previously evaluated the cranial morphology of Japanese preterm infants in the first month after birth and observed that the incidence of dolichocephaly was higher in preterm infants than that in full-term infants, with the risk factors for dolichocephaly being female sex, delivery via cesarean section, and use of artificial ventilation [13]. To our knowledge, no reports examining the evolution of cranial morphology in preterm infants have been published in Japan. Therefore, the present study aimed to investigate temporal changes in cranial shape among preterm infants up to 6 months of age and to explore the relationship between their development and cranial shape at 6 months of age. Hypotheses from clinical impressions were that (1) in preterm infants with dolichocephaly at discharge, dolichocephaly will continue for a long time, (2) preterm infants are at high risk for DP, and (3) cranial shapes are not associated with developmental quotient (DQ).

## 2. Materials/Subjects

### 2.1. Ethics

The ethics committee of Kasukabe Medical Center approved the protocol for this prospective follow-up study (approval numbers: 2020-020, 2021-007; approval dates: 9 September 2020, and 5 May 2021, respectively). Because porTable 3D scanners are not recognized as medical equipment in Japan, all participants’ parents or custodians provided written informed consent for the use of 3D scanners and their participation in the study.

### 2.2. Participants

Participants were preterm infants who were delivered before 37 weeks of gestation and were hospitalized in our NICU between April 2020 and March 2022. Admission to the NICU at our facility has been restricted to infants born at 30 weeks of gestation or later. Therefore, this investigation was limited to preterm infants born at 30–36 weeks of gestation. Only Japanese infants were considered in this investigation. Participants with chromosomal diseases, intraventricular hemorrhage (Papile grade ≥ 3), or periventricular leukomalacia were excluded from the analysis. The participants did not receive any intervention, including instructions pertaining to repositioning, but were cared for as usual during this investigation.

Full-term infants from our previously reported database were used as the controls [14,15]. Participants in the control group were infants delivered after 37 weeks of gestation who attended our facility at 1 month of age between April 2020 and April 2021, and underwent 3 measurements at 6 months of age. The exclusion criteria were consistent with those of the present study.

### 2.3. D Data Collection and Analysis

A 3D scanner (Artec Eva; Artec Inc., Luxembourg) was used to scan the heads of the participants. The heads of participants were held by a guardian or medical provider during the scan. The hair was covered with an elastic wig cap so that it did not affect the scanning. The scanned 3D data were stored in Standard Triangular Language file format.

### 2.4. Reference Plane

**Figure 1:**
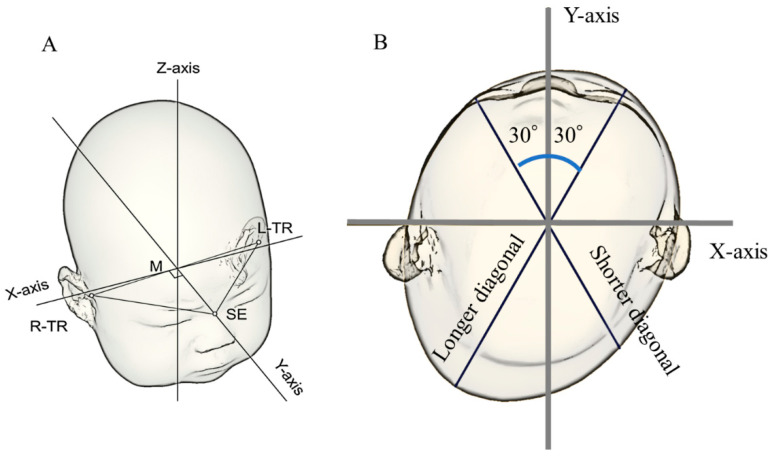
Three-dimensional images. (**A**) Illustration of how the reference plane (Level 0) and X- and Y-axes were determined. (**B**) Cross-sectional view of the measurement plane (Level 3 plane). The X-axis, Y-axis, and 30° diagonals were used to quantify cranial asymmetry and cranial vault asymmetry index. Abbreviations: M, midpoint between the tragions; L-TR, left tragion; R-TR, right tragion; SE, sellion; TR, tragion.

The acquired 3D data were analyzed using an image processing software (Artec Studio; Artec Inc., Luxembourg) and the original image processing program of the Japan Medical Company (Tokyo, Japan). Figure 1, which was adopted from our previous report, shows the approach adopted to establish these parameters [15]. The reference plane was defined as the surface connecting the three sites of the sellion (SE; deepest part of the nasal root) and both tragions (TR; upper edge of the ear tragus) (Level 0). The midpoint between the left and right TRs (M) was determined using this program. The Y-axis was established as the line connecting the locations SE and M. The line perpendicular to the Y-axis at point M was determined to be the X-axis on the Level 0 plane (Figure 1A) [16,17].

### 2.5. Measurement Plane and Symmetry-Related Parameters

The program generated ten equal cranial cross-sections from Level 0 to the summit of the head. The height of each cross-section was calculated by dividing the distance between the Level 0 plane and the top of the cranium into ten equal portions. The Level 3 plane was designated as the measurement plane [16,18], as shown in the cross-section in Figure 1B. The cranial length (CrL) and cranial width (CrW) were measured as the lengths of the measurement plane in the Y-axis and X-axis directions, respectively.

The cephalic index (CI) was calculated as CrW/CrL (%) [19]. Cranial asymmetry was defined as the difference between the longer and shorter axis lengths at 30° from the measurement plane’s Y-axis on the left and right sides [19]. The cranial vault asymmetry index (CVAI) was calculated as cranial asymmetry/shorter axis length (%) [19]. Since cranial asymmetry is underestimated in preterm (small-bodied) infants, CVAI was used to assess DP. Japanese dolichocephaly was defined as CI < 79.2% and brachycephaly was defined as CI > 93.8% [20]. Japanese DP was defined as CVAI > 5% [15,21].

### 2.6. Study 1: Cranial Shape in Preterm Infants over Time

For the longitudinal study, CI and CVAI were measured using a 3D scanner at 1 (T1), 3 (T2), and 6 months (T3) postnatal age. The Shapiro–Wilk normality test was used to determine whether each parameter was normally distributed and to perform any necessary analysis. A longitudinal study was conducted using repeated-measures analysis of variance (ANOVA) or Friedman’s test, and comparisons between time points were performed using Bonferroni correction. The prevalence of dolichocephaly, brachycephaly, and DP was similarly examined for significant differences using Cochran’s Q test and McNemar’s test with Bonferroni correction. Similarly, the progress of preterm infants was compared to that of full-term infants from a previously reported database. The preterm and full-term groups were compared using the Mann–Whitney *U* test, Student’s *t*-test, and Fisher’s exact test.

### 2.7. Study 2: Sex Differences in Cranial Shape in Preterm Infants

Sex differences in CI and CVAI at each time point in the preterm group were examined using the Mann–Whitney *U* test and Student’s *t*-test, respectively.

### 2.8. Study 3: Examination of the Relationship between Development and Cranial Shape at T3

The Enjoji Scale of Infant Analytical Development (Enjoji Scale) [22,23] was used simultaneously with a 3D scan at T3 and development was assessed at the corrected age in months. The association between DQ and either CI or CVAI was examined using Pearson’s product-moment correlation or Spearman’s rank correlation. Sex differences in DQ scores were also examined. All statistical calculations were performed using EZR statistical software (64-bit version 1.61; Saitama Medical Center, Jichi Medical University, Saitama, Japan) [24].

## 3. Results

During the course of this study, 65 Japanese preterm infants underwent 3D scanning at T1 at our hospital. However, as 30 individuals dropped out, 35 infants were assessed at T2. Subsequently, 9 infants dropped out; hence, 26 infants were assessed at T3. Overall, 26 infants who completed all evaluations were included in the present study. The male to female ratio was 1:1. Of these participants, 3 (11.5%) began to undergo cranial helmet therapy for DP after the third measurement (T3).

The full-term group, which comprised 88 healthy Japanese participants from a database previously examined for cranial growth over time at 1–6 months of age, was used as the comparison group [14,15].

### 3.1. Characteristics of the Participants

The characteristics of the preterm and full-term infants are summarized in Table 1. In the preterm group, the average gestational age was 34.7 weeks (range: 30 weeks and 5 days to 36 weeks and 6 days) and the average birth weight was 2114 g (range: 1385–3190 g). Cesarean section, multiple births, and use of artificial ventilation were more common in the preterm group, as reported in a previous study [13].

At each measurement, the chronological age was 35.6 ± 4.4 days for T1, 102 ± 13 days for T2, and 202 ± 16 days for T3. The corrected age, modified by the expected date of delivery (40 weeks and 0 days), was −4.7 ± 11days, 64.5 ± 14days, and 164 ± 16days for T1, T2, and T3, respectively. The mean corrected age at T3 was approximately 5.5 months.

### 3.2. Study 1: Cranial Shape in Preterm Infants over Time

#### 3.2.1. Changes in the CI and Prevalence of Dolichocephaly and Brachycephaly

The progression of CI and the prevalence of dolichocephaly and brachycephaly are shown in Table 2. Because the CI was normally distributed at all time points in the Shapiro–Wilk normality test, a statistical analysis was performed for a normal distribution. The CI in preterm infants was examined over time using repeated-measures ANOVA. Because the Mauchly sphericity test was not rejected (*p* = 0.09), the *p*-values were not corrected. The CI in the preterm group increased significantly over time: 77.2% for T1, 82.9% for T2, and 85.4% for T3. There was a significant increase in the overall course and between the time points (*p* < 0.01 for each time point). A comparison of the 2 groups using Student’s *t*-test revealed a significantly lower CI in the preterm group at each time point. At T3 also, CI value was significantly lower for the preterm group compared to the full-term group (85.4% vs. 89.0%; *p* < 0.01)

The prevalence of dolichocephaly showed significant improvement, particularly at T1–T2 (73.1% to 34.6%, *p* = 0.01) in the preterm group. In addition, at T3, the prevalence of dolichocephaly in the preterm group did not differ significantly from that in the full-term group (15.4% vs. 4.5%, *p* = 0.08). At T3, there was no significant difference in the prevalence of dolichocephaly between the two groups, although there was a significant difference in the CI values.

In the preterm group, there were no infants with brachycephaly at T1 and only 3 (11.5%) at T3. The prevalence of brachycephaly was not significantly different between the groups at any time point.

#### 3.2.2. Changes in the CVAI and Prevalence of DP

The progress of CVAI and prevalence of DP are presented in Table 3. Since the CVAI at T3 was distributed nonparametrically (*p* < 0.01, Shapiro–Wilk normality test), nonparametric methods were used to examine the CVAI. Regarding the progression of CVAI in the preterm group, the Friedman test revealed no statistically significant differences in the overall progression or between time intervals: 4.8% at T1, 5.7% at T2, and 3.3% at T3 (*p* = 0.23). Furthermore, the Mann–Whitney *U* test revealed no significant difference in CVAI values between preterm and full-term infants at any time point. Similarly, no significant change in the prevalence of DP was observed over time: 50% at T1, 53.8% at T2, and 34.6% at T3 (*p* = 0.17). The preterm and full-term group had the same prevalence of DP; there was no significant difference at any time point.

### 3.3. Study 2: Sex Differences in Cranial Shape in Preterm Infants

Sex differences in the CI and CVAI at each time point are shown in Table 4. There were no sex differences in CI and CVAI at any time point.

### 3.4. Study 3: Relationship between Development and Cranial Shape at T3

**Figure 2:**
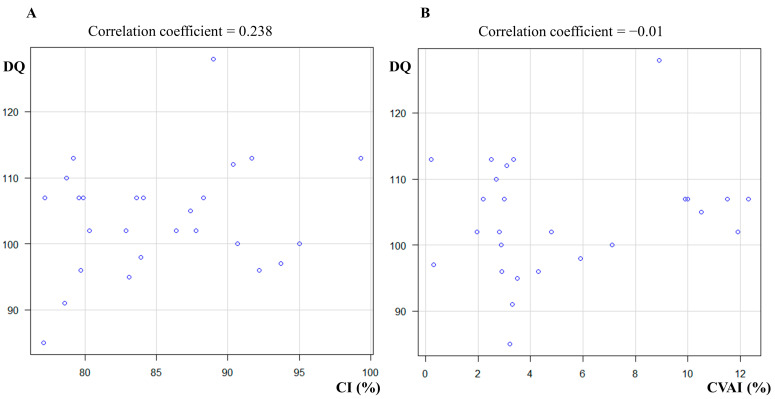
Correlations between CI, CVAI, and DQ scores (**A**) Correlations between CI and DQ at T3. (**B**) Correlation between CVAI and DQ at T3. Abbreviations: CI, cephalic index; CVAI, cranial vault asymmetry index; DQ, developmental quotient.

The average developmental indices measured at 6 months of chronological age in the group comprising 26 preterm infants were as follows: total DQ, 103 ± 8.6; motor, 109 ± 12; social, 102 ± 8.4; and language, 101 ± 10. Furthermore, the mean head circumference at 6 months of age in the preterm group was 427.6 ± 11.5 mm. Because the total DQ was normally distributed (*p* = 0.39 in the Shapiro–Wilk normality test), Pearson’s product-moment correlation was used for the comparison of CI, whereas Spearman’s rank correlation was applied for the comparison of CVAI. The correlation coefficient between the total DQ and CI at T3 was 0.238 (95% confidence interval: −0.164 to 0.572, *p* = 0.24; Figure 2A), whereas the rank correlation coefficient between the total DQ and CVAI at T3 was −0.01 (95% confidence interval: −0.396 to 0.379, *p* = 0.96; Figure 2B). No significant correlation between total DQ and cranial morphology was discovered at 6 months of age. Furthermore, there was no significant correlation between head circumference and DQ at 6 months of age (rank correlation coefficient,0.114; 95% confidence interval, −0.286 to 0.48; *p* = 0.58). No significant sex differences were found in DQ (male/female = 105/102 [mean value]; *p* = 0.40).

Similarly, the total DQ at T3 showed no correlation with either the CI or CVAI at any time point (Table 5). This study found no correlation between cranial morphology and growth in preterm infants during the first 6 months of life.

## 4. Discussion

The present study investigated the relationship between the course of cranial shape change and development in a group of preterm infants up to 6 months of age. The main findings of this study are as follows: (1) dolichocephaly improved over time; (2) preterm and full-term infants exhibited no significant difference with respect to DP; and (3) DQ did not correlate with cranial shape.

### 4.1. Dolichocephaly

Previous studies have reported that preterm infants in other countries were susceptible to dolichocephaly [2,3,4], and that the cranial shape of infants with dolichocephaly at the time of discharge from the NICU improved by 6 months of age [6,7], which is consistent with the findings of the present study. This study also revealed that the prevalence of dolichocephaly in 6-month-old preterm and full-term infants was comparable. Clinically, preterm infants appear to maintain a flattened long head on both sides for a long time. However, in this study, the prevalence of dolichocephaly at 6 months old was not significantly different from that in full-term infants. This may be because this study did not include extremely preterm infants born before the gestational age of 28 weeks, and future studies with long-term follow-up of extremely preterm infants may yield different results. Another factor is the presence of racial bias regarding the prominence of dolichocephaly in preterm infants, because Japanese infants have brachycephaly [20]. Additionally, it is possible that the CI did not express the clinically perceived difference in shape (oval or rounded rectangle viewed from above) because it was simply expressed as the CrL-to-CrW ratio. Therefore, a new index must be established to assess cranial morphology in preterm infants. In previous reports, the female sex was listed as a risk factor for dolichocephaly [3,13]; however, no sex difference was found in our present study. The low number of participants may have been a factor.

### 4.2. Plagiocephaly

Some studies have shown that preterm infants are at risk of DP [3,8], whereas another study reported no difference in the risk between preterm and full-term infants [9]. Contrary to our hypothesis, in this study, neither the CVAI values nor the prevalence of DP differed from those in full-term infants. The Japanese are known to have a high prevalence of DP [15,21]; therefore, the prevalence of DP among controls may also be a factor. In full-term infants, CVAI peaked at T2 and considerably improved from T2 to T3 [15]. The preterm infants examined in the present study also showed a trend similar to that observed in the full-term infants; however, no significant differences were noted.

In the current study, 3 infants (11.5%) required cranial helmet therapy. As 4.8% of mature infants were introduced in the progress study [13,14,15], preterm infants increasingly required helmet therapy; however, the difference was not significant (*p* = 0.18). The small number of participants included in this study may have prevented statistically significant differences, and the high prevalence of DP in the comparison group comprising full-term infants may have led to a discrepancy in the results between this study and reports from other countries. Future multicenter studies involving very preterm infants, including a larger number of participants, are necessary.

In full-term infants, the male sex has been reported to be at a higher risk for DP [25]. In our previous report, we found a significant sex difference in CVAI among preterm infants but no significant difference in the prevalence of DP [13]. In the present study, we found no significant differences in CVAI. A larger study on sex differences in DP is necessary.

### 4.3. Relationship between the DQ and Cranial Shape

In this study, total DQ was not significantly correlated with DP or dolichocephaly, as hypothesized. Infants with DP have been reported to have developmental disorders [10,11,12]; however, only a few reports have indicated an association between dolichocephaly and development in preterm infants [4]. The results of this study provide new insights into this topic.

However, it cannot be denied that the corrected age of 5.5 months for preterm infants in this study was too premature for development assessment. Furthermore, even when developmental delay was observed, it was difficult to determine whether it resulted from premature birth or cranial geometry. Additionally, many participants in this study had a low risk of cranial deformity; therefore, CI and CVAI might show no correlation with DQ at T3. The association between developmental assessment at a corrected age of 1 year and the worst CVAI and CI values should be examined in future studies that include larger populations to allow for multivariate analyses.

### 4.4. Cranial Shape-Correcting Devices

Cranial helmet therapy and the use of a simple pillow to prevent DP have been reported in full-term infants [21,26,27]. In an intervention study, Uchio et al. used a simple pillow for the cranial shape of preterm infants and showed that the intervention group exhibited an improved cranial shape at a corrected age of 6 months compared to the control group and achieved significantly higher Bayley cognitive scale scores and fine motor scores [28]. Since the present study was a follow-up study of preterm infants, no association with developmental cranial shape was found; however, it is possible that better development could be expected with interventions. Further investigations on the use of cranial shape-correcting devices in preterm infants from an early age are necessary. In November 2022, the Food and Drug Administration dissuaded the use of pillows for cranial shape correction [29]. In the future, it is necessary to consider preventive strategies using new devices that are not simple pillows.

### 4.5. Limitations

The present study had some limitations. First, it evaluated only a small number of participants from a single institution; recently, most preterm infants born after 30 weeks have good outcomes owing to good perinatal and neonatal care. This would be a limitation for outcome correlation because a low DQ could be rare in a patient population with limited case numbers. Furthermore, the developmental assessment of 6-month-old infants is sketchy, and many cases need to be considered to assess mild developmental delays. Therefore, future studies should include larger numbers of participants. Second, this study was limited in terms of its development assessment because international evaluation was not possible. Based on this study, future research conducted at multiple institutions is necessary to draw a complete conclusion.

## 5. Conclusions

We described temporal changes in the cranial shape of preterm infants and showed that their development did not correlate with cranial shape. The fact that the dolichocephaly of preterm infants improved over time and that the DP was not significantly different from that of full-term infants is cause for optimism regarding the outcome of the cranial shape in preterm infants. However, this study did not include extremely preterm infants, which suggests that future research is required.

## Figures and Tables

**Table 1:** Participant characteristics.

	Preterm Infants	Full-Term Infants	
N	26	88	
Male	13 (50)	45 (51.1)	
Maternal age, years	34.1 ± 4.8	34.0 ± 5.4	
First birth	9 (34.6)	42 (47.7)	
Delivery method			^†^
Vaginal	5 (19.2)	44 (50)	
Cesarean section	21 (80.8)	34 (38.6)	
Vacuum or forceps	0 (0)	10 (11.4)	
Cephalic fetal presentation	21 (80.8)	82 (93.2)	
Multiple pregnancies	5 (19.2)	4 (4.5)	^†^
Gestational age, weeks	34.7 ± 1.9	38.6 ± 1.3	^†^
Birth weight, g	2114 ± 486	3019 ± 320	^†^
Birth head circumference, cm	31.0 ± 1.9	33.7 ± 1.4	^†^
Mechanical ventilation	7 (26.9)	0 (0)	^†^

The data are presented as the average ± standard deviation or as a number (percentage). ^†^ *p* < 0.05.

**Table 2:** Changes in the cephalic index and prevalence of dolichocephaly.

					*p*-Value			
A		T1	T2	T3	Whole	T1–T2	T2–T3	T1–T3
Cephalic index, %	Preterm infants	77.2 ± 6.1	82.9 ± 7.1	85.4 ± 6.1	<0.01	<0.01	<0.01	<0.01
	Full-term infants	84.7 ± 4.5	88.1 ± 5.6	89.0 ± 5.8	<0.01	<0.01	<0.01	<0.01
	*p*-value	<0.01	<0.01	<0.01				
B		T1	T2	T3	Whole	T1–T2	T2–T3	T1–T3
Prevalence of dolichocephaly, N (%)	Preterm infants	19 (73.1)	9 (34.6)	4 (15.4)	<0.01	0.01	0.22	<0.01
	Full-term infants	10 (11.4)	5 (5.7)	4 (4.5)	0.04	0.55	1.00	0.34
	*p*-value	<0.01	<0.01	0.08				
C		T1	T2	T3	Whole	T1–T2	T2–T3	T1–T3
Prevalence of brachycephaly, N (%)	Preterm infants	0 (0)	2 (7.7)	3 (11.5)	0.17	-	1.0	-
	Full-term infants	1 (1.1)	16 (18.2)	19 (21.6)	<0.01	<0.01	1.0	<0.01
	*p*-value	1.0	0.24	0.40				

The cephalic index is presented as the mean ± standard deviation, whereas the prevalence of dolichocephaly and brachycephaly are presented as a number (%). T1: first measurement at almost 1 month old; T2: second measurement at almost 3 months old; T3: third measurement at almost 6 months old.

**Table 3:** Changes in the cranial vault asymmetry index and prevalence of deformational plagiocephaly.

					*p*-Value			
A		T1	T2	T3	Whole	T1–T2	T2–T3	T1–T3
CVAI, %	Preterm infants	4.8 (2.3–5.9)	5.7 (2.5–9.1)	3.3 (2.8–8.5)	0.23	0.15	0.17	0.55
	Full-term infants	5.0 (3.0–7.0)	5.8 (3.0–7.9)	4.6 (2.5–6.8)	<0.01	0.13	<0.01	0.38
	*p*-value	0.24	0.77	0.91				
B		T1	T2	T3	Whole	T1–T2	T2–T3	T1–T3
Prevalence of DP, N (%)	Preterm infants	13 (50.0)	14 (53.8)	9 (34.6)	0.17	1.00	0.22	1.00
	Full-term infants	43 (48.9)	49 (55.7)	38 (43.2)	0.03	0.63	0.03	1.00
	*p*-value	1.00	1.00	0.50				

The cranial vault asymmetry index is presented as the median (interquartile range), whereas the prevalence of deformational plagiocephaly is presented as a number (%). CVAI, cranial vault asymmetry index; DP, deformational plagiocephaly; T1, first measurement at almost 1 month old; T2, second measurement at almost 3 months old. T3, third measurement at almost 6 months old.

**Table 4:** Sex differences in CI and CVAI.

		T1	T2	T3
CI	Male	77.6 ± 6.6	82.3 ± 7.7	85.1 ± 7.2
	Female	76.8 ± 5.8	83.5 ± 6.7	85.6 ± 5.1
	*p*-value	0.76	0.67	0.85
CVAI	Male	5.2 (3.2–6.3)	5.3 (2.3–10.0)	3.2 (2.9–10.0)
	Female	3.5 (2.0–5.5)	6.0 (3.3–7.3)	3.5 (2.8–5.9)
	*p*-value	0.40	0.94	0.80

The cephalic index was presented as the mean ± standard deviation, and the cranial vault asymmetry index was presented as the median (interquartile range). CI, cephalic index; CVAI, cranial vault asymmetry index; T1, first measurement at almost 1 month old; T2, second measurement at almost 3 months old. T3, third measurement at almost 6 months old.

**Table 5:** Correlation between developmental quotient at T3 and cranial shape at each time point.

A: CI			
	Correlation coefficient	95% confidence interval	*p*-value
CI at T1	0.267	−0.134–0.593	0.19
CI at T2	0.224	−0.178–0.563	0.27
CI at T3	0.241	−0.164–0.573	0.24
**B: CVAI**			
	Rank correlation coefficient	95% confidence interval	*p*-value
CVAI at T1	−0.271	−0.596–0.130	0.18
CVAI at T2	−0.093	−0.464–0.305	0.65
CVAI at T3	−0.010	−0.396–0.379	0.96

CI, cephalic index; CVAI: cranial vault asymmetry index. T1, first measurement at almost 1 month old; T2, second measurement at almost 3 months old. T3, third measurement at almost 6 months old.

## Data Availability

The data presented in this study are available on request from the corresponding author.
